# Understanding Probabilistic Cognitive Relaying Communication with Experimental Implementation and Performance Analysis

**DOI:** 10.3390/s19010179

**Published:** 2019-01-06

**Authors:** Amith Khandakar, Amr Mahmoud Salem Mohamed

**Affiliations:** 1 Electrical Engineering Department, College of Engineering, Qatar University, Doha-2713, Qatar; 2 Computer Science and Engineering Department, Qatar University, Doha-2713, Qatar; amrm@qu.edu.qa

**Keywords:** cognitive relaying, GNU Radio, probabilistic relaying, USRP2

## Abstract

Efficiently use of the limited wireless spectrum can be achieved by cooperative cognitive relaying, where secondary users (SUs), who do not pay for the licensed spectrum and have better channel condition to the primary users (PUs) destination, can help the PU by relaying their traffic. A systematic approach of implementing a Cooperative Cognitive Relaying framework using USRP2 is proposed in this paper, which could be used for practical experiments on cognitive radio applications. Two probabilities are introduced in the experiment in the paper and their effect on the PU and SU performance are studied and analyzed. The two probabilities are: (1) Probability of Admission, which controls the PU data that would be allowed by SU in their PU data queue (which could be relayed by SU later) and (2) Probability of Scheduling, which controls the selection of queue at the SU (PU relay data queue or the SU data queue) and the data of the selected queue would be relayed by SU during an idle time slot. Finally, the practical results from the varying of the introduced probabilities on the performance of PU and SU are verified with the simulation results. A very interesting result is found from the practical experiment where it is seen that increasing probability of scheduling of the PU packets at the SU is always in favor of the SU as opposed to the PU in terms of both throughput and delay.

## 1. Introduction

As wireless communication has become the de facto standard for the growing and diverse demands of users worldwide, there is a need to use the spectrum as efficiently as possible to accommodate future innovations. The wireless spectrum can be considered as an exhaustible resource shared by multiple users, and standardized regulatory authorities govern the sharing of this spectrum. One of the popular techniques that provides a unique solution to the problem is smart or cognitive radio (CR). Concerns of how to efficiently use the limited and sometimes underutilized licensed spectrum [1] motivates research in the cognitive radios concept. CR tries to make use of the periods when the spectrum is unused, known as spectral holes, and in the process also make sure to not hamper the valid users who are paying for it, termed the primary users (PUs) [2,3]. The spectrum is idle during the spectral holes period. The reason for these spectral holes is the bursty nature of the sources where at certain times there is a lot of data to be sent and at other times there is nothing to be sent. Cognitive radio lets some users makes use of those idle periods. These users do not pay for the spectrum and are termed secondary users (SUs). The SUs try to not overload the already limited wireless spectrum and, whenever possible, assist in increasing the PU’s performance. In other words, efficiency of the spectrum utilization by different users is improved without disturbing the quality of service (QoS) requirements of the PU, who are the legal users as they are paying for the spectrum [3].

In order to efficiently manage the SU’s use of the PU’s licensed spectrum, cooperative communication is needed and it has been widely analyzed [4,5,6,7,8,9]. Cooperative communication in cognitive radio should be beneficial for both SUs, as they can use licensed spectrum without paying for it and for the PUs, as SUs should assist in increasing the PUs’ communication performance. Due to the nature of wireless channels, a single transmission can be heard by dissimilar nodes in the vicinity and data can sometimes be lost between transmitter and the intended receiver. However, the lost data can be successfully received by the dissimilar nodes in the range and with the cooperative communication nature, these dissimilar nodes can in turn relay the lost traffic to the intended receiver. With the inclusion of added security, it can be ensured that the transmission is only heard by the dissimilar nodes and not interpreted. In Refs. [4,10], the authors discuss some cooperative communication protocols such as amplify-and-forward and decode-and forward schemes. Characterization of the performance was done with respect to outrage events and probabilities in Ref. [4] while in Ref. [10] the authors have investigated the transmission power of the relaying nodes and developed a cooperative spectrum sensing scheme with the interference constraint where they have found that the sensing performance depends not only on the interference tolerance level but also on the relay protocol used. In Ref. [11], many partner cooperative transmission protocols are proposed and the performance was analyzed using Zheng-Tse diversity-multiplexing trade-off [12]. In Ref. [13], Ibrahim et al. provided an analysis using the symbol error rate for decode and forward cooperation protocol, which was developed into relay selection mechanism and analyzed in Ref. [14]. Cooperative communication can be considered similar to spatial diversity where multiple nodes in the former is similar to the multiple antennas helping to achieve spatial diversity in single communication link [15,16,17]. In Refs. [18,19], the authors proposed a spectrum-leasing scheme, where the SU can perform either energy harvesting from the radio signal of the PU and later transmit using the harvested energy, and compared them with conventional sensing schemes.

There are many studies on developing cooperative cognitive relaying and comparing the performance. In Ref. [20], power is allocated at the SU with the objective of maximizing the stability or reliability of the fixed throughput of the cognitive link. In Ref. [21], a fraction of the PU bandwidth is allocated to SU transmission based on gain achieved from cooperation. Many protocols, which allow collaboration between a single PU and multiple SU, are studied in Ref. [22] and inspired the writing on dirty paper (WDP) concept which is analogous to sender writing the message on a dirty paper which independent dirt spots of normally distributed intensity and the reader cannot distinguish between the dirt spots and ink marks applied by the writer [23]. Performance gains in terms of stable throughput region and average delay are demonstrated in Ref. [17] and have been used in the experimental setup in this paper. In Ref. [24] the authors have studied the energy efficient power allocation schemes for OFDM based cognitive radio networks. There are studies [25,26], where the authors have worked on the security concerns in cognitive radio networks. To the best of the author’s knowledge, in all the studies done in cognitive radio network or cooperative communication in cognitive radio network, there has not been a practical implementation of the system and using the practical implementation to analyze the performance. All of the studies are based on simulation and numerical results.

Cooperation in cognitive radio networks not only allows SUs to transmit their own data during the idle time slots but also promises to help relaying lost PU packets, thus confirming that cooperation in cognitive radio networks is beneficial in all possible ways [27]. Retransmissions by the PU are reduced as the SU’s assist PU’s to deliver their packets to the destination. This provides more idle slots that can be used by SUs for their own use, and this process is termed as cognitive relaying. However, this is subjected to SU having a better channel to PU destination; else, even the relayed traffic by the SU would not reach the PU destination. Cognitive relaying helps in making more time slots available for SU’s to transmit it data after fulfilling the demands of the PUs.

In this paper, a detailed description of the practical implementation of a Cooperative Relaying MAC protocol is done, it is implemented and the results from the implementation is discussed and analyzed. The implemented MAC protocol relies on the SU having two queues. One of the SU queues is used for queuing its own generated packets and the other queue is for storing the dropped PU packets so that it can relay them and assist the PU in successfully transmitting the data to the destination. Investigational experimental setup involving USRP and SDR platforms such as LabVIEW, Matlab, and Gnu Radio have been done for applications such as spectrum sensing and cooperative relaying [28,29] but they did not show the step by step MAC protocol implementation. This paper will also further investigate the feasibility of improving the cooperative relaying performance, benefiting both the PU and SU, with the help of two introduced conditional probabilities.

Contrasting to the general assumption of always allocating priority to the relaying queue at SU, i.e., the queue that stores PU packets, an investigation on the performance is done by introducing 2 probabilities, one for the admission of PU packets in the relay queue of SU and another for the SU transmitting the PU packets whenever it finds an idle slot [17], these probabilities are termed as Probability of admission (*P_a_*) and Probability of Service (*P_s_*) or Relaying Queue selection probability, respectively. The introduced probabilities implies that SU will service the data in its own queue with (1 − *P_s_*) probability whenever it finds an idle time slot. Using the performance metrics (i) delays of PU, (ii) delays of SU, and (iii) throughput of PU packets, and with the help of tuning capability of the introduced probabilities, it is possible to enhance the performance of either of them (it can be controlled to prioritize PU performance) or both. Based on the user end applications running on the SU and PU, which determines the allowable delay, it is possible to analyze the performance of SU and PU by tuning the introduced probabilities. All the results are confirmed with simulation results and some of the results are analyzed with the theoretical work of the same authors in Ref. [17]. The simulation results help in verifying the practical implementation of the cooperative cognitive relaying framework. The practical implementation tries to verify the control in the cooperative cognitive relaying performance using the degrees of freedom provided by the two introduced probabilities. The main contributions of this work are summarized as follows:

- We have established an investigational background for reviewing cognitive relay scenario, making use of policies for scheduling and admission.
- A MAC layer protocol, with systematic instructions, for reliable and synchronized communication between PU Transmitter, Receiver and the Relaying SU is implemented.
- We analyze how the admission and scheduling probabilities could be tuned to improve SU performance while not affecting the PU’s performance.
- The feasibility of practical implementation of a cognitive relaying scenario with introduced probabilities is verified and the results compared with theoretical values from the authors’ previous work [17].

The main novelty of the paper is the systematical approach provided for developing a cooperative cognitive relaying framework. The working of the framework is compared with simulation results. The advantage of authors previous work in developing the theoretical model in Ref. [17] have helped in analyzing and confirming some of the interesting results from the practical experiments. The rest of this paper is organized as follows: Section 2 presents the system model along with the test setup description. Section 3 discusses the results obtained from the test setup and compares it with theoretical results in Ref. [17] and finally the conclusions are drawn in Section 4.

## 2. System Model and Description of the Test Setup

In order to have efficient spectrum sharing, i.e., not causing interference to existing traffics, SUs need to have some information of the PU’s traffic and based on the information, cognitive radio approaches can be categorized into: Underlay, Overlay and Interweave [30]. The description of each of the categories is stated below:

Underlay: Cognitive radios constrained to cause minimal interference to noncognitive radios. This means the SU’s have the least amount of information of the PU’s traffic and the complexity is the least.

Interweave: Cognitive radios find and exploit spectral holes to avoid interfering with noncognitive radios.

Overlay: Cognitive radios overhear and enhance noncognitive radio transmissions. This category requires the maximum information of the PU traffic compared to other categories and so is the complexity involved in it.

In this paper, the overlay category of cognitive radio approach is implemented, as overhearing by SU is required for the efficient implementation of the Cooperative Cognitive relaying with some introduced probabilities.

### 2.1. Description of the Test Setup

**Figure 1:**
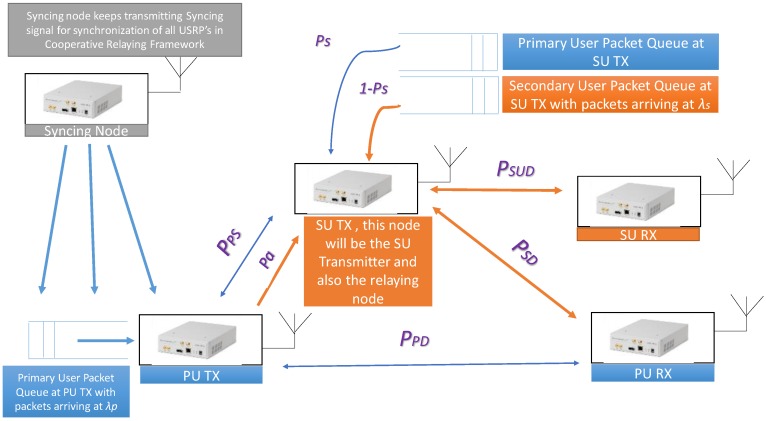
Summarizing the Test Setup.

The detailed description of the test setup is shown in Figure 1. It is explained elaborately below:

- Syncing Node: This node synchronizes the time slots of the Secondary User’s (SU’s), Primary User Transmitter (PU TX) and Primary User Receiver (PU RX), by transmitting Sync packets periodically, as easily implementable Time Division Multiple Access MAC protocol has been used.
- PU TX: This node is the Primary User which is transmitting.
- PU RX: This node is the Primary User which is receiving.
- SU TX: This node is the Secondary User transmitter which performs cooperative cognitive relaying based on the introduced probabilities.
- SU RX: This node is the Secondary User receiver for the transmitted SU TX, secondary users, packets.

In the paper, the authors assume that the channel gain and the noise processes are not random, the movement of the nodes does not alter the channel quality and the only possible reason for the packet to be decoded incorrectly is due to the channel quality. Receiving an entire packet correctly would mean a successful transmission else, it is categorized as erroneous packet and discarded. As Time Division Multiple Access is used, the SU transmits if and only if it finds any time slot idle ensuring that the system cannot have collisions for multiple nodes transmitting at the same. Since Carrier Sense Multiple Access (CSMA) MAC protocol is utilized, channel impairment (such as shadowing, fading, additive noise and signal attenuation) is the only reason attributing to packet loss. Whenever the signal to noise ratio of the channel between the transmitter and receiver is below a minimum threshold (below which correct decoding of packets is not possible) then packet loss occurs which is the channel outage event.

Let *P_PS_*, *P_PD_* and *P_SD_* represent the probability of successful transmission between the PU TX and SU TX, PU TX and PU RX, and the SU TX and PU RX, respectively. Throughout the paper, it is assumed that *P_PD_* < *P_SD_*, as we want the SU to act as a cooperative relay which can happen only if it has a better channel to the destination compared to the PU. In the practical implementation, this is achieved by setting the numerical value of *P_PD_* less than *P_SD_* in the scripts running on the USRP’s. It has to be noted that there is a single queue in the PU TX where the PU packets generated by PU TX is stored. There are two queues in the SU TX, one for storing the PU TX packets that are to be relayed and the other is for storing the SU packets generated by the transmitter of SU communication link.

A detailed summary of the probabilities used in the paper and in the experimental setup is stated below and also in Table 1. These which will also be used to get the simulation results, which would confirm the correctness of the practical implementation:

- *P_PS_* is probability of successful transmission between PU TX and SU TX.
- *P_SD_* is the probability of successful transmission between SU TX and PU RX.
- *P_PD_* is probability of successful transmission between PU TX and PU RX.
- In order to compare the performance of a cooperative cognitive relaying we need to assume that *P_PS_*, *P_SD_* > *P_PD_*, which implies that the communication link between PU TX and PU RX via SU is comparatively stronger/better than the communication link between PU TX and PU RX directly. This is the best scenario for a cooperative cognitive relaying. In other words, the values set to the probabilities will emulate the channel condition and it is set to values to emulate better channel condition between PU TX and SU TX, SU TX and PU RX compared to PU TX and PU RX. This is why it is assumed that *P_PS_* = 0.95, *P_SD_* = 0.95 and *P_PD_* = 0.70.
- *P_SUD_* is the probability of successful transmission between SU TX and SU RX. These parameter is set to a value to emulate the good channel condition between SU TX and SU RX, i.e., *P_SUD_* = 0.9.
- *P_a_* is the probability of admission, which determines the probability whether SU TX will allow the PU TX relay data (PU data that is not successfully received by the PU RX) in the PU packet Queue at SU TX. In other words, it can be the degree of Cooperative Cognitive Relaying permissible.
- *P_s_* is the probability of scheduling/servicing PU packets, which determines the probability that SU TX during an idle time slot, when it can use the channel, can relay the packets in the PU packet queue at SU TX. This implies that (1 − *P_s_*) is the probability of scheduling/servicing the packets in the SU packet queue at SU TX.
- *λ_p_* denotes the rate at which packets arrive at the PU TX queue according to Poisson distribution. It is set to a value to avoid the overflowing of the queue, i.e., *λ_p_* = 0.4
- *λ_s_* denotes the rate at which packets arrive at the SU TX queue according to Poisson distribution. It is set to a value to avoid the overflowing of the queue, i.e., *λ_s_* = 0.4.

### 2.2. MAC Description

The details of the MAC protocol implemented is stated below. The results from the variation of the introduced probabilities on the performance of the PU and SU will be analyzed in the results section:

- If the receiver PU successfully interprets the transmitted packet from PU then it broadcasts an ACK. This packet exits the system. If it is not received by the receiver PU but was successfully received by the SU TX then the SU TX buffers the packet in its PU packet queue with the admission probability (*P_a_*) or discards it (1 − *P_a_*).
- Once the SU TX buffers a packet, it sends back an ACK to declare successful reception of the PU TX’s packet. It becomes the obligation of the SU TX to deliver the packet to the destination.
- In the worst case when the packet is neither successfully received by the PU RX nor by the SU TX, it is retransmitted by the PU TX in the next time slot.
- When a time slot does not have any PU activity, the SU TX uses it to transmit either a packet from Primary User packet Queue at SU TX (with probability *P_s_*), refer Figure 1, or from Secondary User packet Queue at SU TX (with probability (1 − *P_s_*)).
- Whenever a packet is not exited from the system upon reception of ACK then it is kept at queue (sender either PU or SU) for later retransmission.
- In case there is no packet to be sent by the sender either PU or SU then the channel remains idle.

The proposed policy of the MAC is non-work-conserving for the simplicity of implementation. A system is considered work conserving if it does not remain idle whenever it has packets [8]. If the working scheme is understood then it can be noticed that in the test setup, whenever there is an idle slot the SU may have something to transmit either the PU TX data that it would relay or its own data and if neither queue have any packet, the slot remains idle. It is done for reducing the complexity of the implementation and the performance of such an implementation being work conserving is a future work, which would require Time Division Multiple Access (TDMA) not being implemented as packets could be generated during the middle of an idle time slot. In such a situation, it might not be transmitted even if there is an idle slot, as it has to wait for the next time slot, which is against the work conserving queueing theory [8]. Implementation of work conserving queuing theory with appropriate MAC could be future work. It should also be noted that by adding encryption to the packets SU will not be able to decode the PU Data but will only help in relaying it.

### 2.3. States and Their Description

#### 2.3.1. PU TX States

**Figure 2:**
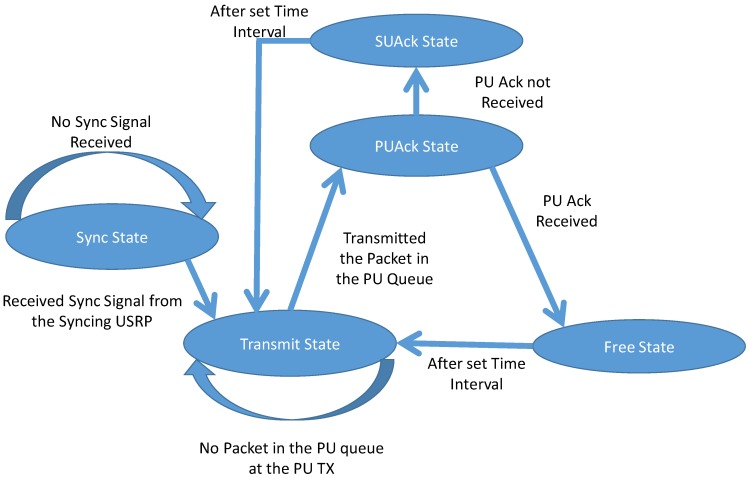
State Diagram of the PU TX.

PU TX can be in three of the four states as shown in Figure 2 and Table 2. The three states exclude the default sync state. The default sync state is the state that all the USRPs entering the cooperative cognitive relaying framework has to be in for getting synchronized to the other USRPs in the framework. In Figure 2 and Table 2, it can be seen that the PU TX has four States and it can be in any three of them, based on state diagram and MAC protocol design, stated briefly in the previous section. Each state is allocated same time-period which depends on the time required for transmitting packets. The time- period of three states constitutes the frame duration. Each frame denotes a packet being transmitted by either PU TX or SU TX. The states move from one state to another due to the criteria stated in the state diagram or due to time out of the interval.

*Transmit State*: Whenever there is packet generated in the PU queue with a rate *λ_p_*, PU TX tries to transmit it to the receiver. The transmission of PU data happens in this state. After transmission of single packet, the PU TX moves to the next state *PUAck*. PU TX stays in this state until there is a packet to be transmitted in its queue.

*PUAck State*: After the transmission of packet PU TX waits for acknowledgement of receipt from PU RX, and this happens in this state. After this state, the PU TX can go to either *Free State or SUAck State*.

*Free State*: Having received the acknowledgement from PU RX, there is no activity in this state and thus it will be in the Free State. The PU TX will exit the packet from its queue as it was received successfully. This state marks the end of the frame and more packets, if available in PU TX queue, is transmitted in the next frame.

*SUAck State*: After the timeout of PUAck state and not receiving any acknowledgement from PU RX, PU TX waits for the acknowledgement from the SU TX which confirms that it has received the PU data and will be transmitting it whenever the channel is free from any PU activity. Thus, it informs the PU TX that it does not have to retransmit and can exit the packet from its queue. For obvious reason, the acknowledgement depends on *P_a_*, which is the probability of admission of PU data in the PU packet queue of SU TX. In case, no acknowledgement is received from the SU TX, which could be due to *P_a_* or the packet was not received by SU TX, then PU TX does not exit the packet from its queue and will retransmit it in the next frame. This state marks the end of the frame and the packet is transmitted in any of the next frames by the SU depending on the activity of the channel or retransmitted by the PU TX in the next frame.

*Sync State*: This state is to synchronize the time slots of all the USRPs-PU TX, PU RX, SU TX and SU RX. A dedicated USRP is always broadcasting the Sync packet to synchronize any new USRP to the time slots of the remaining communicating USRPs in the cooperative cognitive relaying framework. Once synced then the USRPs continue their states as per the protocol and does not have to come back to this state unless it resets.

#### 2.3.2. SU TX States

**Figure 3:**
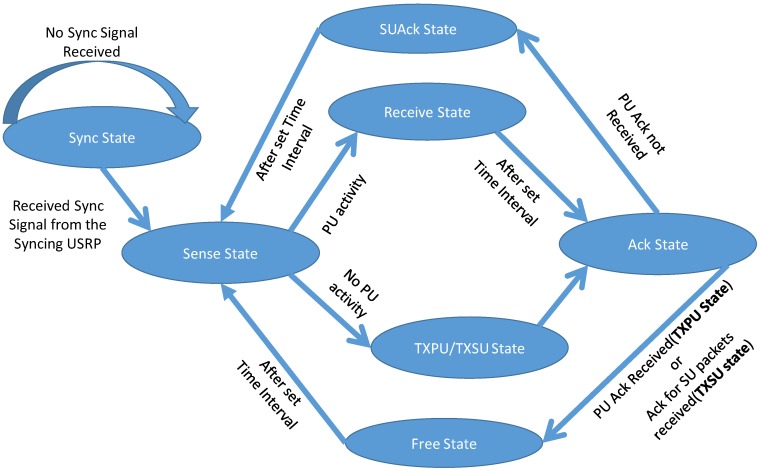
State Diagram of the SU TX.

**Figure 4:**
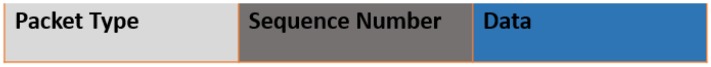
Packet format in the test setup.

SU TX can be in the four states out of the six states as shown in Table 2 and Figure 3. The four states exclude the default sync state. The transition from one state to another depends on the criterion shown in Figure 4 and as per the protocol design stated in the earlier section.

*Sense State*: Based on a sensing algorithm developed in the MAC layer, SU TX senses the spectrum for any PU activity with the help of Packet Type Field, refer Figure 4. The basic energy detection spectrum sensing technique was used for sensing the spectrum for any activity. The python script for spectrum sensing is readily available in GNU Radio, i.e., Software Defined Radio (SDR) platform used in the experiment. Once the spectrum is found to be occupied, the SU TX can look at the packet type field, refer Figure 4, to know whether the activity is due to PU or SU. A predefined number is assigned for PU or SU activity. The SU senses in the initial 30% of the time-period of the first state, after which it moves to either *Receive State* or *TXPU/TXSU State*. The reason for selecting the time period for the sense state is so that SU TX will have enough time to take decision regarding which state to move to and conduct the activity in that state such as receive the PU data or transmit the PU or SU data.

*Receive State*: Upon sensing activity, SU TX moves to this state and stores the PU packet in one of the two queues it has, i.e., PU Packet Queue, depending on *P_a_*.

*TXPU/TXSU State*: If there is no PU activity, then SU TX can use the channel, where it can either transmit its own data with a probability (1 − *P_s_*) or the queued PU Data (which were not acknowledged by PU RX and was allowed based on *P_a_*) with probability of *P_s_*.

As shown in Table 2, the *Sense State* and *Receive State* or *TXPU/TXSU State* collectively has one time-period time slot. After these states, SU enters the *Ack State*.

*Ack State*: In this state, SU TX waits for acknowledgment of the receipt of PU TX data or SU data (as it can transmit SU data in the *TXPU/TXSU* state depending on (1 − *P_s_*)) coming from the PU RX or from the SU RX respectively. Upon reception of acknowledgement, the respective packet exits from the respective queue (either PU Packet Queue or SU Packet Queue). Obviously, it will not receive both the acknowledgement at the same time, as SU is not allowed to use the channel if there is PU activity.

Depending on the previous state SU TX can be in the below next states:

- *Receive State*: If in the previous state, SU TX was in *Receive state* then based on the receipt of acknowledgement of PU data, it transits to either the *Free State* or the *SUAck State*.
- *TXPU State*: If in the previous state, SU TX was in *TXPU/TXSU* state and it has transmitted PU data, then it will transit to either *Free State* or *SUAck State* depending on the reception of acknowledgement from PU RX.
- *TXSU State*: If in the previous state, SU TX was in *TXPU/TXSU* state and it has transmitted SU data then it will only transit to *Free state*. If the acknowledgement of SU packet is received then that particular packet exits the SU data queue of SU TX, else it will be transmitted in the next available opportunity.

*SUAck State*: In case of no acknowledgement received from PU RX after PU activity, stating that the packet was not received by it and SU TX have received it during *Receive State* (depending on *P_a_*), SU TX acknowledges PU TX that it would relay the packet for it whenever the channel is idle from PU’s activity This state marks the end of frame.

*Free State*: In case of acknowledgement received from PU RX or from SU RX (depending on whether PU data was sent by PU TX or SU data by SU TX respectively), there is no activity in this state and thus it will be the Free State. This state marks the end of the frame.

#### 2.3.3. PU RX States

**Figure 5:**
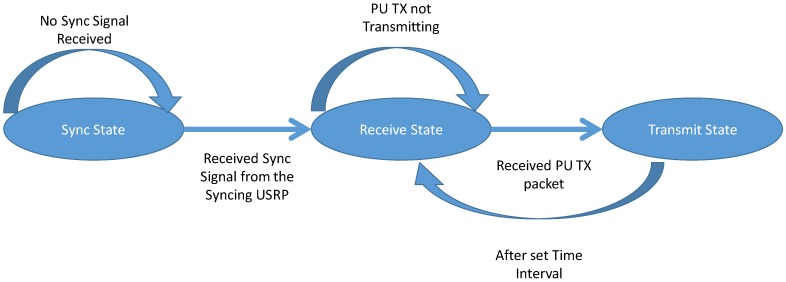
State Diagram of the PU RX.

PU RX can be in the three states as shown in Table 2 and Figure 5 and the three states, that it can be in, excludes the default sync state. The transition from one state to another depends on the criterion shown in Figure 5 and as per the protocol design stated in the earlier section.

*Receive State*: In this state, PU RX waits for the reception of either the PU TX packet. Unlike other states in PU TX and SU TX, the PU RX remains in this state until it receives any packet from either PU TX. After the complete reception of packet or time-period, it moves to the *Transmit State*.

*Transmit State*: After the packet is received from PU TX, it acknowledges the reception of the data packet, which could be heard by all the USRP’s in the framework. In case the packet is not received, then it does not send any acknowledgement and moves to the *receive* state again.

#### 2.3.4. SU RX States

**Figure 6:**
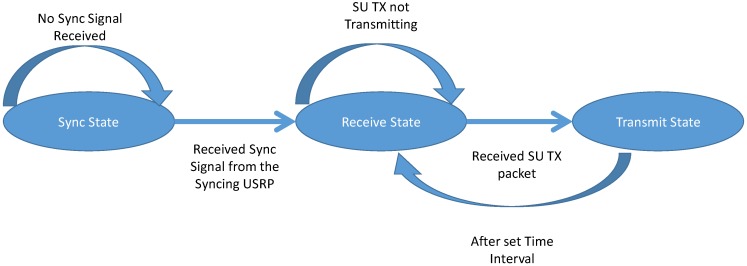
State Diagram of the SU RX.

The states of the SU RX as shown in Figure 6 is similar to the PU RX and is described in the previous section.

## 3. Discussion of Results

**Figure 7:**
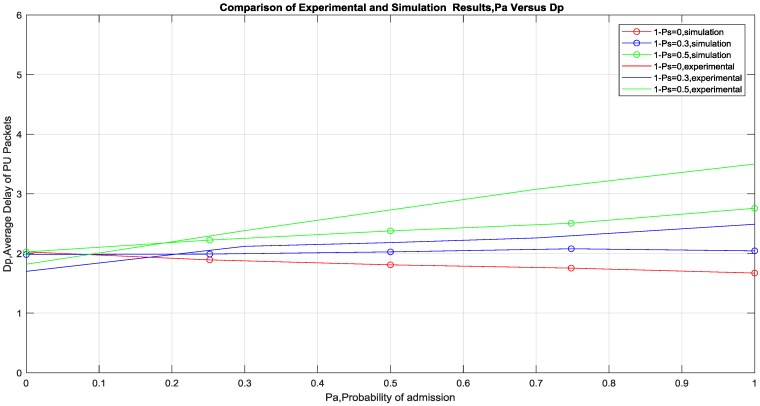
Simulation and experimental results of *D_p_* Versus *P_a_* for varying (1 − *P_s_*).

**Figure 8:**
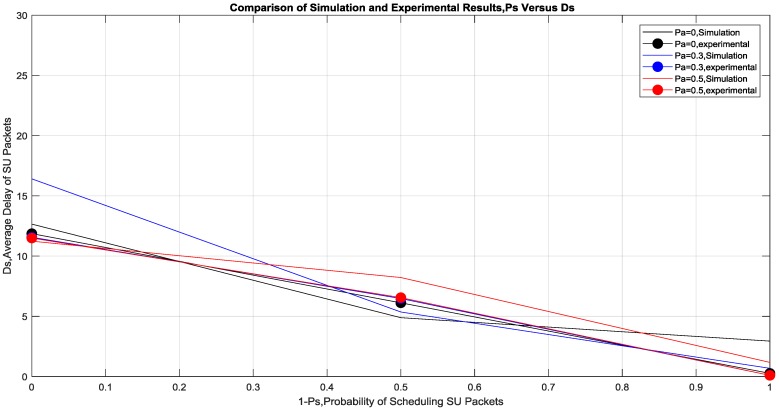
Simulation and experimental results of *D_s_* versus 1 − *P_s_* for varying *P_a_*.

**Figure 9:**
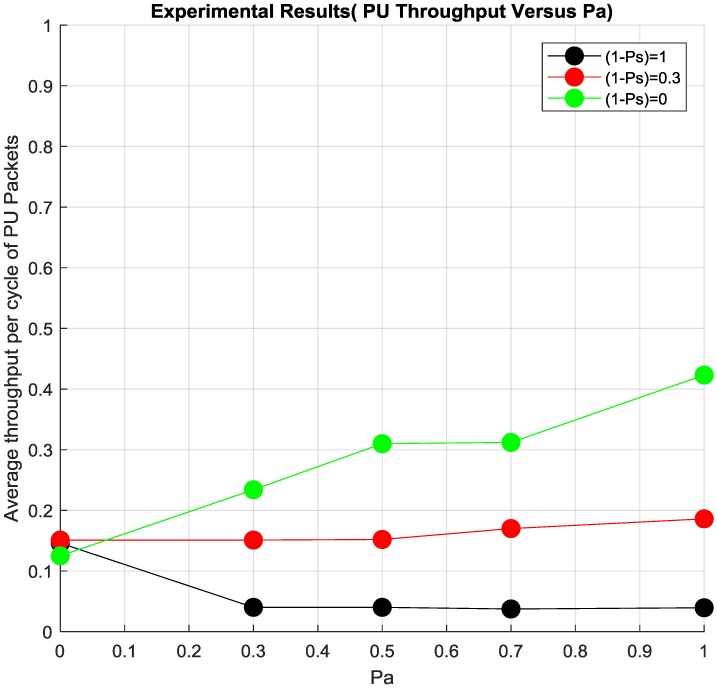
PU packets throughput per cycle.

Performance metrics such as Average Delay of PU packets in the queue of PU TX (*D_p_*), Average Delay of SU packets in the queue of SU (*D_s_*) and Average throughput of PU packets per frame are used in the result shown in Figure 7, Figure 8 and Figure 9. As shown in Figure 7, the experimental results and the simulation results (using the same MAC protocol design) show similar trends where the *Average PU packets Delay* is constant for a particular (1 − *P_s_*) and increases monotonically with *P_a_* for a comparative higher (1 − *P_s_*) and decreases monotonically with *P_a_* for a comparative lower (1 − *P_s_*). In fact, at (1 − *P_s_*) = 0.5, the simulation and practical results completely overlap each other. This could be explained with the reasoning that the benefit of cooperation is inversely proportional to the quality of the channel between the PU TX and PU RX. Since the probability of successful delivery of packets increases due to the better channel quality and thus there is no need of cooperation. It is also found in the result that below a certain (1 − *P_s_*), probability of scheduling SU packets, cooperation is beneficial to PU in terms of Average Delay of PU packets. This is an interesting result as it is not necessary for (1 − *P_s_*) to be very low to be beneficial for the PU. This was further verified with the analytical expression in [17], which estimates this threshold of (1 − *P_s_*), to be given by ((1 − *P_PD_*)/*P_SD_*). This threshold value of (1 − *P_s_*) is found to be around 0.3 in the experimental setup and it can be seen that above this value the cooperation is not beneficial to PU. Whereas below the threshold value it is beneficial to the PU and at the threshold the performance is constant despite the increase of *P_a_*. The results also show the effect of *P_a_* in the performance of the system and it could be used in confirming the numerical value, which should be beneficial to both PU and SU transmission. In order to quantize the effect of *P_a_* further investigation is needed but it could be deduced from the results that it does not have significant degrading effect on the PU performance at lower values.

Figure 8 shows that the *Average SU packets Delay* is decreasing with increasing (1 − *P_s_*), which confirms that SU is always benefits from increasing *P_a_*, as it prevents a lot of retransmission from the PU by relaying the dropped packets for it, which in turn provides it with more idle time slots. These idle time slots can be used by SU for transmitting its own data depending on *P_s_*. The experimental results are very similar to the simulation results. At fixed *P_a_*, SU’s packets have lower delay at higher values of (1 − *P_s_*) as more scheduling of its own data. This confirms the benefit of cooperation for both SU and PU by fine-tuning the probabilities based on the channel quality.

Another interesting finding of Cooperation Cognitive relaying could be seen in Figure 9. The average throughput per cycle for PU packets, which is a measure of the number of PU packets that are received by the PU RX during the whole experiment duration, which was around 1 week. PU benefits with having higher throughput with increasing *P_a_* when (1 − *P_s_*) is set to 1, which means SU should be always relaying the PU Data whenever the channel is free. The throughout is not equal to one or is not perfect as the channel is not perfect as there might be retransmissions even from the SU due to low channel quality.

Similarly, the throughput is worst and steady when there is no cooperation at (1 − *P_s_*) set to 0. Interestingly the throughput is constant and better with *P_a_* at certain (1 − *P_s_*) which is a clear benefit of cooperation and also tuning (1 − *P_s_*) so that it benefits both the SU and PU. This value of (1 − *P_s_*) was later found to be in accordance to Ref. [17] where (1 − *P_s_*) < ((1 − *P_PD_*)/*P_SD_*) i.e., (1 − *P_s_*) < 0.3.

It can be clearly seen in Figure 9, that when (1 − *P_s_*) = 1 (i.e., the probability of scheduling SU packets is 1) then the PU does not benefit from it in terms of Average throughput per cycle of PU packet. In the other hand, when (1 − *P_s_*) = 0 (i.e., probability of scheduling SU packets is 0) then the PU benefits completely from the cooperation as the average throughput per cycle of PU packets increases with Pa. In this particular scenario there is no benefit that the SU gets from this cooperation.

Thus there is a middle way out where at a particular (1 − *P_s_*), the average throughput per cycle of PU packets is more compared to when there is no cooperation. It will also help SU as they can use the spectrum at certain times for transmitting their packets. It should be noted that the difference between the practical and simulation results is due to the fact that both of them are based on different probabilities.

## 4. Conclusions

The paper has demonstrated a practical implementation of a Cognitive Relay environment using USRP2 and Gnu Radio. Certain Probability parameters are introduced to meet the requirements of having a trade-off of Throughput and Delay for both the PU and SU. The findings were verified [17] using the Cognitive Relay framework where increasing (1 − *P_s_*) is always in favor of the SU as opposed to the PU in terms of both throughput and delay. The work provides a systematic approach for cooperative cognitive relaying and validates its working with interesting results. One of the major concerns that could arise in the implementation of such cooperative cognitive relaying is the security issue, where it has to be ensured that the SU can only overhear the information, for sensing purpose only, but not interpret it. There are many current studies conducted in this direction and implementing the security can be a prospect of future work for this paper. Furthermore, the implementation setup does not consider the energy-harvesting scheme in cognitive radio, which is also being invested in recent studies [18]. The implementation does not consider mobility and the scenario involving multiple SU’s. In this scenario, cooperation is needed between the SU’s for selecting the SU with the best channel, to the PU RX, to relay the lost PU data.

## Figures and Tables

**Table 1:** Summary of the probabilities used in the implementation and simulation.

Probabilities	Numerical Value
*P_PS_*, probability of successful transmission between PU TX and SU TX	0.95
*P_SD_* is the probability of successful transmission between SU TX and PU RX	0.95
*P_PD_* is probability of successful transmission between PU TX and PU RX	0.70
*P_SUD_* is the probability of successful transmission between SU TX and SU RX	0.9
*λ_p_* denotes the rate at which packets arrive at the PU TX queue according to Poisson distribution	0.4
*λ_s_* denotes the rate at which packets arrive at the SU TX queue according to Poisson distribution	0.4

**Table 2:** Time slot assignments for the different states.

Name of Slot for Different USRP’s in the Cooperative Cognitive Relaying Framework	Description of Time Slots			
PU TX States	Transmit		PUAck	SUAck (No PU Ack Received)
				Free (PU Ack Received)
PU RX States	Receive (Always in this state and transition after time interval happens only when PU transmission is sensed)		Transmit	Receive (Extra State to synchronize all USRP’s)
SU TX States	Sense	Receive State (PU activity sensed)	Ack (It can be PUAck if PU data is transmitted or it can be SUAck if SU data is transmitted)	SUAck (No PU Ack received)
		TX PU/TX SU (No PU Activity Sensed)		Free (PU Ack or SU Ack Received)
SU RX States	Receive (Always in this state and transition after time interval happens only when SU transmission is sensed)		Transmit	Receive (Extra State to synchronize all USRP’s)
	Time Period of 1 state		Time Period of 1 state	Time Period of 1 state
	Frame Duration			
